# Twelve‐Week Group‐Based Exercise and Nutrition Programs: Feasibility and Preliminary Health Improvements in Disabilities

**DOI:** 10.1111/ggi.70650

**Published:** 2026-07-12

**Authors:** Geon Young Jang, Sunghwan Ji, Ji Yeon Baek, Eunju Lee, Jin Go, Chang Ki Lee, Sang Soo Yu, Ju Jin Jung, Il‐Young Jang

**Affiliations:** ^1^ Division of Geriatrics, Department of Internal Medicine, Asan Medical Center University of Ulsan College of Medicine Seoul Korea; ^2^ Department of Digital Health, SAIHST Sungkyunkwan University Seoul Korea; ^3^ Gohigh Vascular Center Seoul Republic of Korea; ^4^ Goldman Urology Clinic Seoul Republic of Korea; ^5^ HIT Plastic Surgery Clinic Seoul Republic of Korea; ^6^ PyeongChang Health Center and County Hospital PyeongChang Gangwon State South Korea; ^7^ Department of Clinical Research Design and Evaluation, SAIHST Sungkyunkwan University, Seoul, Korea

## Abstract

This single‐arm pilot study evaluated a 12‐week group‐based exercise and nutrition program for 22 community‐dwelling adults with disabilities at a local public health center in rural Korea. The program was feasible and well tolerated, with modest preliminary improvements in physical performance.

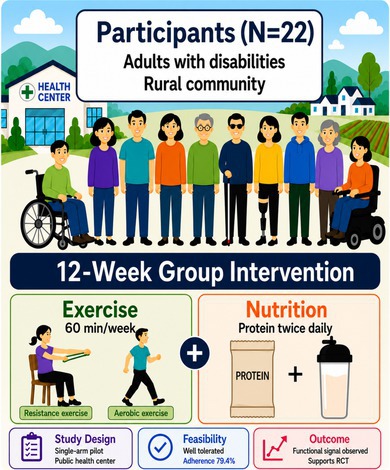

1

In South Korea, disabilities are legally defined and systematically classified under the Welfare Law for Persons with Disabilities, encompassing both physical and mental disabilities. Physical disabilities include external impairments (such as mobility, brain lesions, and sensory disabilities) and internal organ dysfunction, while mental disabilities include intellectual, developmental, and psychiatric conditions. Accordingly, individuals registered under this system are referred to as persons with disabilities, representing a heterogeneous population with diverse underlying impairments and functional limitations. While individualized rehabilitation programs are recommended for this population [1], the implementation of such comprehensive interventions remains challenging in rural areas due to limited healthcare resources [2].

To address this gap, we leveraged a program with established evidence in older adults with frailty and sarcopenia [3]. Disability and these conditions share a bidirectional relationship, whereby frailty and sarcopenia drive functional decline toward disability, while pre‐existing disability in turn accelerates their development [4, 5]. To explore whether this intervention could be effectively delivered in individuals with disabilities, we conducted a single‐arm, community‐based feasibility study among persons with disabilities recruited through a local public health center in rural Korea.

The program consisted of weekly, group‐based exercise sessions, each lasting 60 min, led by experienced community trainers. Each session included a brief warm‐up and cool‐down focused on stretching, with the main exercise component comprising resistance training (30 min) and aerobic exercise (20 min). In addition to supervised exercise, participants received individualized nutritional education at baseline from public health center nutritionists and were provided with a soy protein–based nutritional supplement (13 g protein per 25 g serving), which they were instructed to consume twice daily between meals throughout the intervention period. Exercise attendance was recorded at each session, and adherence to nutritional supplementation was self‐reported by participants at the start of every session. Feasibility and multidimensional outcomes were assessed to explore preliminary signals across physical, functional, and psychosocial domains. Pre–post changes and 95% confidence intervals were estimated using a mixed linear model, with the intervention period as the fixed effect and participants as random effects. This study was approved by the Institutional Review Board of Asan Medical Center, Seoul, South Korea (IRB number: 2024‐0797), and all participants provided written informed consent.

Participants were recruited from PyeongChang Health Center and County Hospital between January and March 2024. A total of 22 registered persons with disabilities enrolled in the group‐based exercise and nutrition program, and all completed the 12‐week intervention. The mean adherence rate to the program was 79.4%, and no intervention‐related adverse events were reported. Across multiple outcome measures, changes were generally observed in a direction consistent with improvement following the intervention (Table 1). Among these, a measurable change was observed in physical performance as assessed by the Short Physical Performance Battery (SPPB), whereas other outcomes showed modest or directionally consistent changes.

**TABLE 1:** Baseline characteristics and changes in outcome measures after the intervention.

Section A. Baseline characteristics	
Characteristics	Value
Age (years)	51.3 ± 14.7
Female, *n* (%)	9 (40.9%)
Body mass index (kg/m^2^)	26.8 ± 4.5
Classification of registered disability, *n* (%)	
Mental disability	11 (50.0%)
External disability	9 (40.9%)
Internal disability	1 (4.5%)
Combined disability	1 (4.5%)
ADL disability, *n* (%)	9 (40.9%)
IADL disability, *n* (%)	17 (77.3%)

Section B. Outcome measures			
Outcome	Baseline	Follow‐up	Mean change (95% CI)
Frailty index (0–1)	0.221 ± 0.126	0.205 ± 0.126	−0.016 (−0.057, 0.025)
SPPB (0–12)	9.5 ± 2.2	10.1 ± 1.9	0.68 (0.18, 1.19)
Gait speed (m/s)	0.85 ± 0.27	0.91 ± 0.28	0.06 (0.00, 0.13)
Grip strength (kg)			
Male	36.5 ± 9.1	36.0 ± 8.6	−0.52 (−1.85, 0.82)
Female	19.1 ± 4.6	20.3 ± 5.3	1.14 (−0.37, 2.66)
Skeletal muscle index (kg/m^2^)			
Male	8.05 ± 1.32	8.19 ± 1.37	0.14 (−0.03, 0.31)
Female	6.16 ± 1.22	6.15 ± 1.17	−0.00 (−0.08, 0.07)
PHQ‐9 (0–27)	3.2 ± 4.0	2.3 ± 2.9	−0.91 (−2.05, 0.23)
EQ‐5D (0–1)	0.81 ± 0.10	0.83 ± 0.09	0.02 (−0.02, 0.06)

*Note:* Baseline characteristics are presented as means ± standard deviations or *n* (%). Outcome measures are shown as means at baseline and follow‐up, with mean changes summarized descriptively as mean differences with 95% confidence intervals.

Abbreviations: ADL, activities of daily living; EQ‐5D, EuroQol 5‐Dimension; IADL, instrumental activities of daily living; PHQ‐9, Patient Health Questionnaire‐9; SPPB, short physical performance battery.

Our pilot study suggests that a 12‐week group‐based exercise and nutrition program can be delivered to individuals with disabilities with good tolerability, high adherence, and no reported adverse events. Although most outcomes did not demonstrate statistically distinguishable changes, improvements in physical performance—specifically SPPB scores and gait speed—reached established thresholds for clinically meaningful change [6, 7], though these findings should be interpreted cautiously given the small sample size and absence of a control group. While changes in other outcomes were modest, their directionally consistent patterns suggest the possibility of multidimensional benefit.

Globally, individuals with disabilities represent a vulnerable population facing substantial barriers to health promotion and preventive care, challenges that are particularly pronounced in rural areas due to limited resources, healthcare facilities, and access to specialized professionals [2, 8]. A key strength of our study lies in its pragmatic use of existing public health center infrastructure. By repurposing a program originally developed for older adults with frailty and sarcopenia, we adapted an evidence‐based intervention to benefit individuals with disabilities without requiring complex, individualized modifications. This approach lowers structural barriers to participation and underscores the potential scalability of community‐based interventions in resource‐limited rural settings.

The heterogeneity of our study population limits the interpretation and generalizability of the findings, as disability type may influence response to the intervention. Nonetheless, this heterogeneity also reflects a strength of the study, as demonstrating feasibility across diverse disability types supports the broader applicability of this intervention in real‐world community settings. A more tailored approach for each disability type might yield stronger and more specific results in future studies. This study provides the preliminary evidence and feasibility data necessary to inform the design of future randomized controlled trials.

## Author Contributions

**Geon Young Jang:** conceptualization, study design, data analysis and interpretation, drafting the manuscript. **Sunghwan Ji:** methodology development and statistical analysis support, critical revision of the manuscript. **Ji Yeon Baek:** data curation, validation, visualization, manuscript review and editing, correspondence. **Eunju Lee:** supervision, critical review and editing of the manuscript, correspondence. **Jin Go:** methodological consultation and study support, critical review of the manuscript. **Chang Ki Lee:** formal analysis and investigation, manuscript review. **Sang Soo Yu:** resources, data validation, manuscript review. **Ju Jin Jung:** data acquisition, investigation, data curation, and critical review of the manuscript. **Il‐Young Jang:** supervision, conceptualization, critical review, and editing of the manuscript. All authors read and approved the final manuscript and agreed to be accountable for all aspects of the work.

## Funding

This research was supported by the Korea Health Industry Development Institute (KHIDI), funded by the Ministry of Health & Welfare, Republic of Korea (grant number: HR20C0026).

## Conflicts of Interest

Jin Go is the Chief Executive Officer of Dr. WELLNESS (Seoul, Korea), the manufacturer of the nutritional supplement used in this study. The supplement had been donated independently of this research project. Dr. WELLNESS had no role in the study design; data collection, analysis, or interpretation; or preparation of the manuscript. All other authors declare no competing interests.

## Data Availability

The data that support the findings of this study are available on request from the corresponding author. The data are not publicly available due to privacy or ethical restrictions.
